# Immunotherapy in elderly head and neck cancer patients: a systematic review and meta-analysis

**DOI:** 10.3389/fonc.2024.1395838

**Published:** 2024-05-10

**Authors:** Viola Salvestrini, Saverio Caini, Melissa Scricciolo, Michael Saerens, Heleen Bollen, Pierluigi Bonomo, Francesca Caparrotti, Luigi Lorini, Marc Oliva, Maria Urbanowicz-Nijaki, Petr Szturz

**Affiliations:** ^1^ Radiation Oncology Unit, Oncology Department, Azienda Ospedaliero Universitaria Careggi, Florence, Italy; ^2^ Cancer Risk Factors and Lifestyle Epidemiology Unit, Institute for Cancer Research Prevention and Clinical Network, Florence, Italy; ^3^ Radiation Oncology Unit, Ospedale dell’Angelo, Venice, Italy; ^4^ Department of Medical Oncology, Ghent University Hospital, Ghent, Belgium; ^5^ Department of Oncology, Laboratory of Experimental Radiotherapy, and Radiation Oncology, UZ Leuven, Leuven, Belgium; ^6^ Radiation Oncology Department, Clinique Générale Beaulieu - Swiss Medical Network, Geneva, Switzerland; ^7^ Medical Oncology and Hematology Unit, IRCCS Humanitas Research Hospital, Milan, Italy; ^8^ Department of Medical Oncology, Institut Català d’Oncologia (ICO) L´Hospitalet, Barcelona, Spain; ^9^ Department of Medical Oncology, Institut d’Investigació Biomèdica de Bellvitge (IDIBELL), Barcelona, Spain; ^10^ Cancer Pathology Unit, Greater Poland Cancer Center, Poznan, Poland; ^11^ Department of Oncology, University of Lausanne (UNIL) and Lausanne University Hospital (CHUV), Lausanne, Switzerland

**Keywords:** immunotherapy, elderly, head and neck cancer, locally advance head and neck cancer, recurrent and metastatic head and neck cancer

## Abstract

**Introduction:**

Over the past years, there has been a growing interest in the role of immunotherapy in locally advanced (LA) and recurrent and metastatic head and neck squamous cell carcinoma (R/M HNSCC). High-quality data from prospective trials are lacking for the elderly subpopulation. This systematic review and meta-analysis aims to review the efficacy and safety of immunotherapy in older patients.

**Methods:**

A systematic literature search was conducted. Randomized clinical trials providing outcome data on a subgroup of elderly (>65 years old) were available for meta-analysis. Primary outcomes of interest were OS and PFS for efficacy analysis.

**Results:**

Seven studies were included in the systematic review and four in the efficacy analysis. The pooled analysis of OS and PFS showed a consistent benefit (HR 0.78 and 0.91, respectively).

**Conclusions:**

Immunotherapy may be an effective and well-tolerated treatment option in the elderly population, but more prospective and randomized data are needed.

**Systematic Review Registration:**

PROSPERO (CRD42022333891).

## Introduction

Head and neck squamous cell carcinoma (HNSCC) is the seventh most common malignancy in the world with over 300,000 cancer deaths per year worldwide (1). More than 25% of HNSCC patients are older than 65 years old at the time of diagnosis (2). Due to demographic changes, the number of elderly affected by this disease will continue to rise, particularly in the Western population, in which HNSCC is expected to increase by over 60% by the year 2030 (3). Apart from other factors, such as tobacco and alcohol abuse, advanced age alone is a risk factor for HNSCC (4). Important differences exist between elderly and younger HNSCC patients. A significant proportion of elderly HNSCC patients is vulnerable and frail and tends to have multiple comorbidities. In addition, lower performance status, worse nutritional status, and impaired organ function compared with younger patients play an important role in treatment decision-making. Furthermore, older patients are prone to developing severe acute and late toxicity after treatment of locally advanced HNSCC (5, 6).

More than half of patients with locally advanced (LA) HNSCC will develop local and/or regional recurrences with or without distant metastases within 3 years of treatment (7). The prognosis of patients with recurrent and/or metastatic HNSCC (R/M HNSCC) is generally poor, with a median overall survival (OS) of 8 to 15 months (7). Since most R/M HNSCCs are not amenable to curative therapy, patients mostly receive systemic treatment. Over the past years, there has been a growing interest in the role of immunotherapy in R/M HNSCC. In this regard, the KEYNOTE-048 trial evaluated pembrolizumab, an anti-PD-1 monoclonal antibody, as a first-line treatment option for R/M HNSCC (8). Significant OS benefit for pembrolizumab monotherapy in patients with PD-L1-positive tumors and for pembrolizumab with chemotherapy in all patients was reported, albeit without improvement in progression-free survival (PFS) or objective response rate (ORR). The survival benefit of PD-1 inhibitors was retained in elderly patients, which is an interesting finding considering the fact that for chemotherapy regimens several studies have described a decreasing effect on survival with increasing age (9). However, since the median age of patients in the KEYNOTE-048 trial was 62 years, the elderly population remains underrepresented. The benefit of PD-1 inhibitors in the second-line treatment of R/M HNSCC was proven in the CheckMate 141 and KEYNOTE-040 studies, reporting a significant survival improvement with nivolumab and pembrolizumab, respectively (10, 11).

Even though HNSCC affects mainly older patients, high-quality data from HNSCC prospective trials are lacking due to underrepresentation or exclusion of this subpopulation from clinical trials (12). As a consequence, data on the safety and efficacy of immunotherapy in elderly adults with R/M HNSCC are still limited. The generally mild toxicity profile of PD-1 inhibitors suggests a safe and effective administration, as already shown for elderly patients with non-small-cell lung and urothelial cancers (13, 14). The KEYNOTE-048 trial reported a favorable safety profile for pembrolizumab monotherapy compared with the EXTREME regimen. In addition, nivolumab delayed time to quality of life deterioration in CheckMate 141 for the overall patient population (10).

Despite the encouraging results of PD-1 inhibitors in the overall HNSCC population, the limited evidence in the elderly subpopulation poses a challenge in clinical decision-making, rendering directives for clinical decision-making limited. This systematic review and meta-analysis aims to comprehensively review the efficacy and safety of immunotherapy in elderly patients with LA and R/M HNSCC.

## Methods

### Search strategy

A comprehensive literature search of PubMed/MEDLINE, Cochrane, and Embase databases was conducted. The literature search was performed in December 2022 (2000 to December 2022) using the keywords “recurrent; metastatic; locally; advanced; head and neck cancer; oral cavity; pharynx; oropharynx; hypopharynx; larynx; immunotherapy; immune checkpoint inhibitor.” The review followed the Preferred Reporting Items for Systematic Reviews and Meta-Analyses (PRISMA) statement (15). Titles and abstracts were screened by two independent researchers (VS and MS) after removing duplicates. Across the articles identified in the initial systematic review, if studies were phase II/III randomized controlled trials (RCTs) and met the inclusion criteria (**Supplementary Material 1**), they were included in the meta-analysis. Moreover, studies were eligible for meta-analysis if they provided a hazard ratio (HR) and a corresponding measure of statistical uncertainty [e.g., 95% confidence intervals (CI), standard errors, variance, or exact *p*-values]. Data extraction was completed by two (FC and MS) independent reviewers to ensure consistency and accuracy.

### Outcomes

The co-primary endpoints of the present analysis were the OS and the PFS of elderly patients with LA and R/M HNSCC of the oral cavity, oropharynx, hypopharynx, or larynx. We defined the elderly population as the subgroup of patients older than 65 years old. The secondary endpoints of the meta-analysis were the safety of single-agent immunotherapy and the health-related quality of life (HRQoL) of the elderly subgroup. Missing data on outcome or toxicity were requested from the corresponding author. To estimate the benefit of immunotherapy on the basis of PD-L1 expression was not part of our investigation due to the lack of data for the elderly subgroup of patients. We adopted the Cochrane tools to assess the risk of bias for randomized and non-randomized controlled trials (16). Two independent reviewers (MO and LL) assessed the risk of bias in each trial, and a third author (VS) was consulted in case of disagreements. We used the GRADE (Grading of Recommendations, Assessment, Development and Evaluations) strategy (17) to assess certainty in the body of evidence for an outcome. The full methodology and statistical analysis can be found in **Supplementary Material 1**.

## Results

### Study characteristics

**Figure 1:**
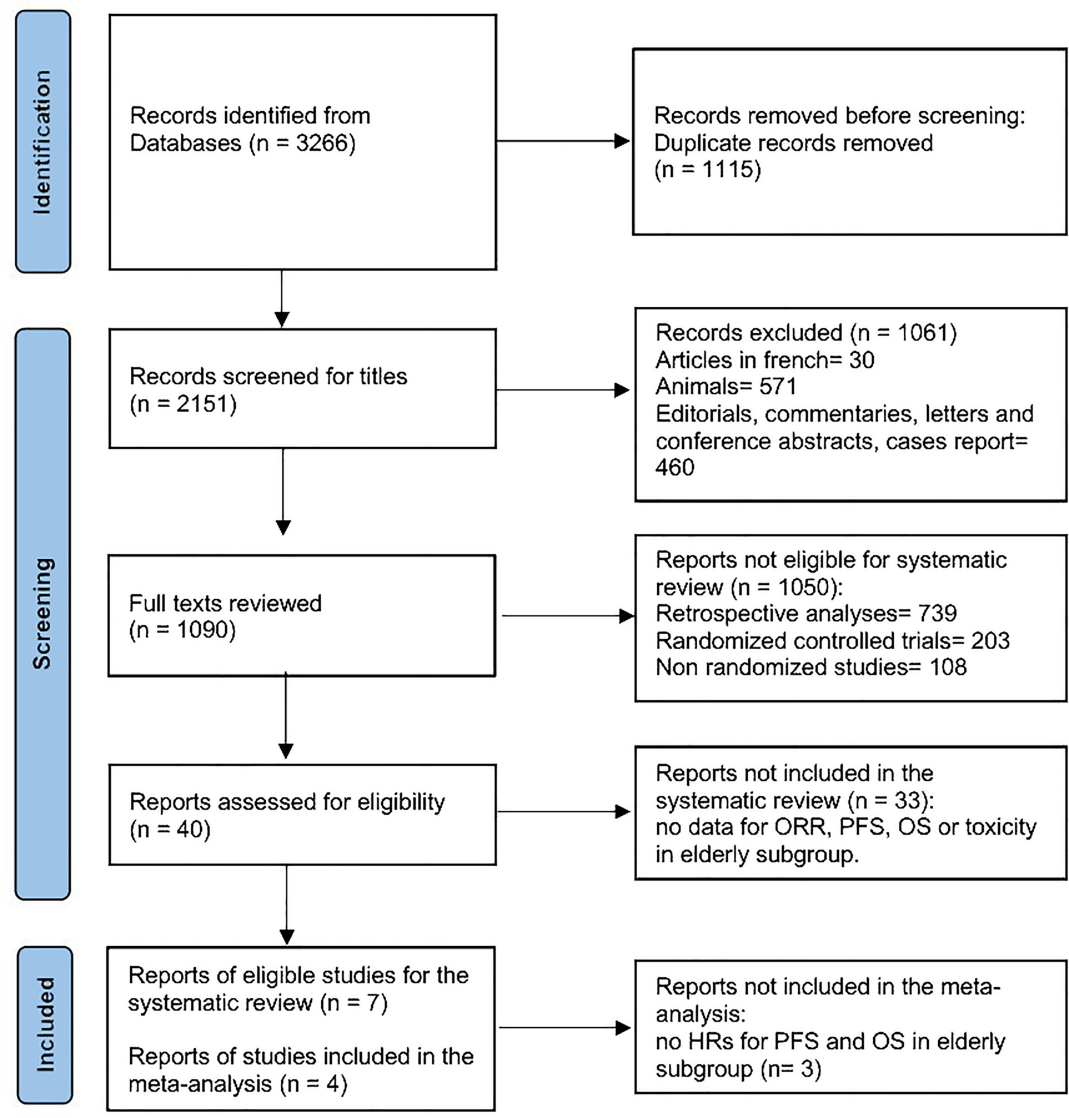
The PRISMA flow diagram depicting the search strategy in the systematic review literature search.

This systematic literature search identified 3,266 articles (**Figure 1**). After adjusting for duplicates, 2,151 articles were screened, 1,090 full texts were reviewed, and 40 reports were assessed for eligibility. Seven articles met all inclusion criteria for systematic review (8, 11, 18–22) and four were finally included in the meta-analysis (8, 11, 18, 19). Three studies were not included in the meta-analysis due to the lack of HRs for OS and PFS in the elderly subgroup (20–22). All the seven included trials were evaluated for toxicities. A total of 4,056 patients were included in the review, with 901 patients aged more or equal to 65 years old. Median age ranged from 57 to 62 years, and the follow-up period ranged from 5 to 43 months.

### Survival outcomes

The main efficacy outcome characteristics are summarized in **Table 1**.

**Table 1:** Overview of the included studies in locally advanced and recurrent/metastatic HNSCC treated with immunotherapy.

Author *Study name*	Setting	Median age, years	65+ patients/all patients	Study design	Intervention (65+ patients/all patients)	Control (65+ patients/all patients)	Primary outcome	Secondary outcome	ORR all patients, % (95% CI)	ORR 65+ patients, % (95% CI)	PFS all patients, HR (95% CI)	PFS 65+ patients, HR (95% CI)	OS all patients, HR (95% CI)	OS 65+ patients, HR (95% CI)
**Lee** *JAVELIN-100*	LA HNSCC	60	202/697	Phase 3 RCT 1:1	Avelumab + CRT (102/350)	Placebo + CRT (100/347)	iPFS	OS, ORR, DoR, pCR, LRF, DMD, safety, PK, PROM, tissue biomarkers	Intervention: 259/350, 74% (69–79) vs. control: 260/347, 75% (70–79) Odds ratio 95% CI: 0.95 (0.66–1.35)	NA	1.21 (0.93–1.57)	1.16 (0.73–1.85)	1.31 (0.93–1.85)	NA
**Saba** *CheckMate 141*	R/M HNSCC, second line	60	113/361	Phase 3 RCT 2:1	Nivolumab (68/240)	IC^a^ (45/121)	OS	PFS, ORR	Intervention: 32/240, 13.3% vs. control: 7/121, 5.8% Odds ratio 95% CI: 2.49% (1.07–5.82)	Intervention ≥65 [10/68 (14.7%, 7.3–25.4)] vs. Control ≥65: [2/45 (4.4%, 0.5–15.1)]	0.89 (0.7–1.13)	0.74 (0.49–1.11)	0.70 (0.51–0.96)	0.75 (0.51–1.12)^b^
**Cohen** *KEYNOTE-040*	R/M HNSCC, second line	60	163/495	Phase 3 RCT 1:1	Pembrolizumab (82/247) ≥65 to <75 (63/247) ≥75 (19/247)	IC^a^ (81/248)	OS	OS PD-L1 >1, safety, PFS, ORR, DoR, TTP	Pembrolizumab: 36/247 IC: 25/248	NA	0.96 (0.79–1.16)	NA	0.80 (0.65–0.98)	≥65 to <75 0.57 (0.37–0.87) ≥75 1.13 (0.42–3.02)
**Burtness** *KEYNOTE-048*	R/M HNSCC, first line	61	180/882	Phase 3 RCT 1:1:1	A: pembrolizumab (NA/301) B: pembrolizumab + platinum + 5FU (NA/281)	C: cetuximab + platinum + 5FU (NA/300)	OS, PFS	Safety, ORR, 6m PFS, 12m PFS, QoL	A: pembrolizumab (OR 51/301) B: pembro + cht (OR 100/281) C: cetuximab − cht (OR 108/300)	NA	A vs. C: 1.34 (1.13–1.59) B vs. C: 0.92 (0.77–1.10)	NA NA	A vs. C: 0.85 (0.71−1.03) B vs. C: 0.77 (0.63–0.93)	A vs. C: 0.82^c^ (0.61–1.10) B vs. C: 0.55^d^ (0.40–0.75)
**Segal** *NCT01693562*	R/M HNSCC, second line or more	57	21/62	Phase I/II single arm	Durvalumab (21/62)	/	Safety	ORR, DCR, DoR, PFS, OS	4/62 [6.5% (1.8–15.7)]	2/21 [9.5% (1.2–30.4)]	6m PFS: 11.9% 12m PFS: 11.9%	NA	6m OS: 62.4% 12m OS: 38%	NA
**Ferris** *EAGLE*	R/M HNSCC, second line	60	222/736	Phase 3 RCT	A: durvalumab (71/240) B: durvalumab + tremelimumab (73/247)	C: SoC^e^ (78/249)	OS	12-, 18-, and 24-month OS and PFS, mPFS, ORR, DoR, safety	A: 41/240 17.9% (13.3–23.4) B: 45/247 18.2% (13.6–23.6) C: 17.3 (12.8–22.5)	NA	A vs. C: 1.02 (0.84–1.25) B vs. C: 1.09 (0.90–1.33)	NA NA	A vs. C: 0.88 (0.72–1.08) B vs. C: 1.04 (0.85–1.26)	NA^f^ A: <65: 79.3% [134/169] 65–74: 73.2% [41/56] ≥75: 73.3% [11/15] B: <65: 83.9% [146/174] 65–74: 82.5% [52/63] ≥75: 80% [8/10] C: <65: 80.1% [137/171] 65–75: 81.3% [52/64] ≥75: 71.4% [10/14]
**Psyrri** *KESTREL*	R/M HNSCC, first line	61	NA/823	Phase 3 RCT 2:1:1	A: durvalumab + tremelilumab (NA/408) B: durvalumab (72/204)	C: cetuximab + platinum + 5FU (extreme) (74/207)	OS (B vs. C) in PD-L1 high (TPS >50% or IC >25%)	OS, PFS and ORR, DoR in all comers for B vs. C and A vs. C; safety, tolerability	A: 35/204 (17.2%) B: 90/413 (21.8%) C: 101/206 (49%)	NA	NA	NA	A vs. C: 1.03 (0.83–1.27) B vs. C: 1.04 (0.87–1.25)	NA^f^: A: NA B: <65 83.3% [110/132] 65–74: 90.1% [55/61] ≥75: 100% [11/11] C: <65: 110/133 (82.7%) 65–74: 85.2% [52/61] >75: 75% [9/12]

LA HNSCC, locally advanced head and neck cancer; R/M HNSCC, recurrent/metastatic head and neck cancer; ORR, objective response rate; DCR, disease control rate; DoR, duration of response; LRF, locoregional failure; DMD, distant metastatic disease; PK, pharmacokinetics; TTP, time to progression; RCT, randomized controlled trial; CRT, chemoradiotherapy; iPFS, investigator-assessed progression-free survival; NA, not available.

a IC: treatment of investigator’s choice: docetaxel, methotrexate or cetuximab.

b HR OS for the age group 65–74 years. HR age group >75 years was 1.13 (0.42–3.02).

c HR OS in 65+ for pembrolizumab versus cetuximab + chemo: all CPS 0.82 [95% CI 0.61–1.10], CPS >1: 0.71 [95% CI 0.51–0.98]; CPS >20: 0.61 [95% CI 0.38–0.99].

d HR OS in 65+ for pembrolizumab + chemo versus cetuximab+chemo: all CPS 0.55 [95% CI 0.40–0.75], CPS >1 0.54 [95% CI 0.39–0.76]; CPS >20 0.67 [95% CI 0.41–1.10].

e SoC: standard of care, investigator’s choice between cetuximab, docetaxel, paclitaxel, methotrexate, 5-fluorouracil, TS-1, or capecitabine.

f Percentage of death by any cause [n patients with event/patients in the age subgroup].

**Figure 2:**
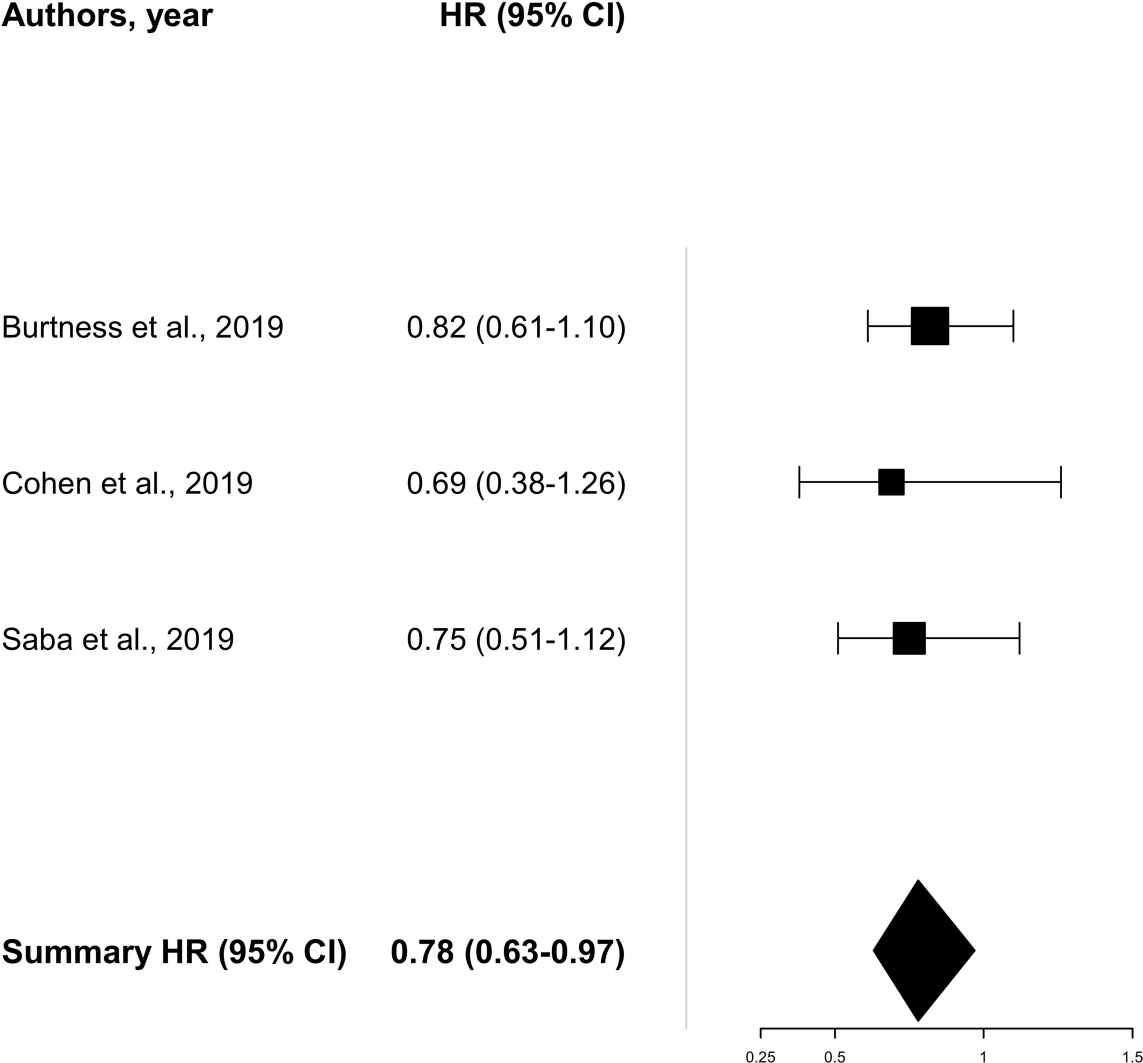
Pooled analysis for overall survival.

**Figure 3:**
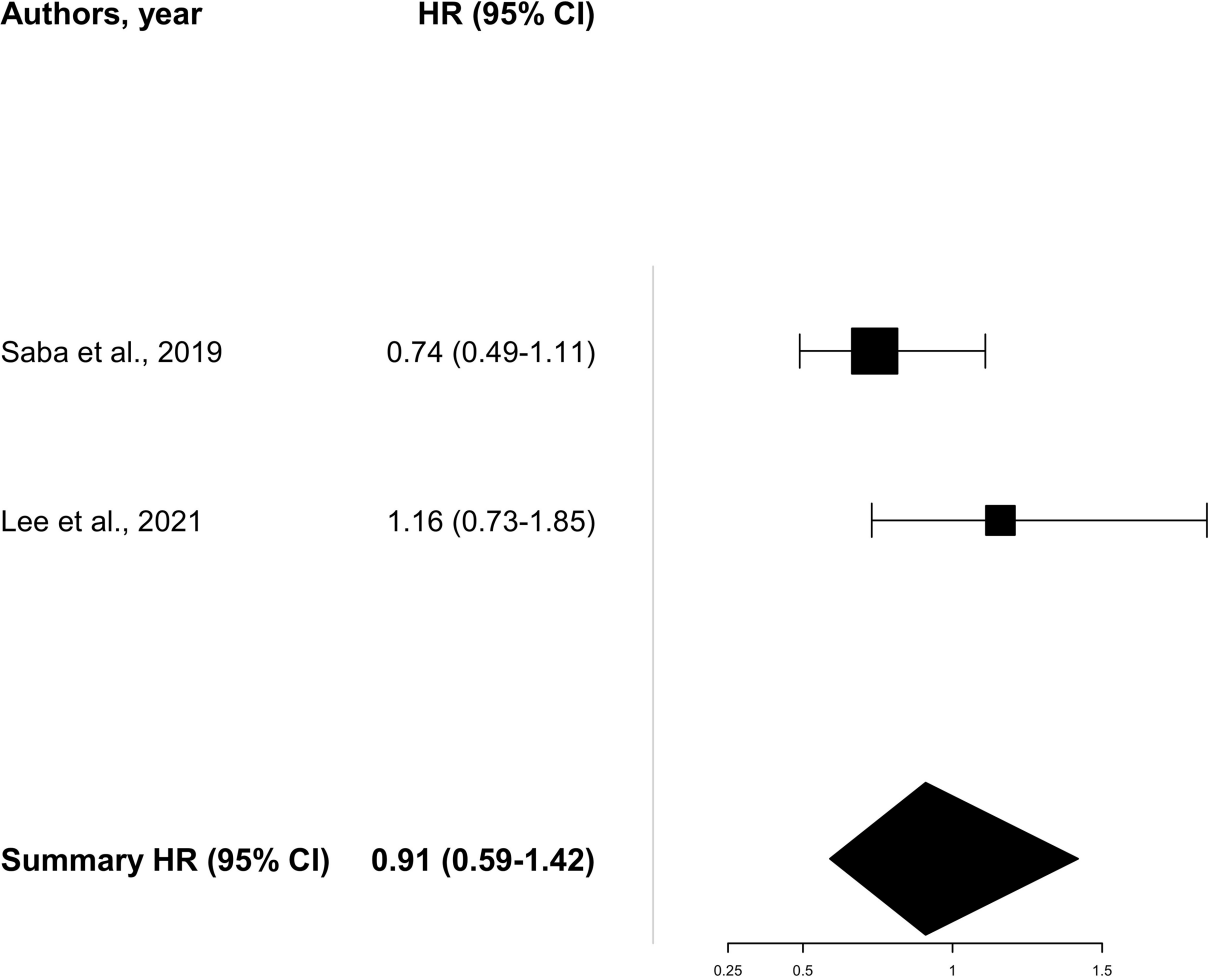
Pooled analysis for progression-free survival.

Due to the missing survival data for patients older than 65 years old, the pooled analysis of OS evaluated only three studies (8, 11, 19) showing a consistent benefit [HR 0.78 (95% CI 0.63–0.97)] with a low level of heterogeneity (*I* ^2^ = 0%). On the other hand, pooled data on PFS (18, 19) including two trials showed an HR of 0.91 (95% CI 0.59–1.42) with an intermediate level of heterogeneity (*I* ^2^ = 51%) (**Figures 2**, **3**). A total of 771 patients (≥65 years old) were included in the meta-analysis (456 for OS and 315 for PFS).

The CheckMate 141 (19) randomized phase III trial confirmed the benefit of nivolumab in the elderly platinum-resistant patients either in PFS [HR 0. 74 (95% CI 0.49–1.11)] or OS [HR 0.75 (95% CI 0.51–1.12)]. The median age was 60 years old, and out of the 113 patients older than 65 years old enrolled in the trial (31.3% of the total population enrolled), 68 received nivolumab and 45 the treatment of investigator’s choice. The ORR for the elderly subgroup was higher in the intervention arm than in the control arm: 14.7% and 4.4%, respectively. The KEYNOTE-040 study was a randomized phase III trial (11) investigating pembrolizumab versus the investigator’s choice as second-line therapy for R/M HNSCC patients. The median age was 60 years old, and 33% of the patients were older than 65 years old. The older population was grouped as follows: 65–74 years old and >75 years old. Of the 247 patients receiving pembrolizumab, 63 (25.5%) were in the first group and 19 (7.6%) in the second group. Hazard ratios of OS were 0.57 (95% CI 0.38–0.87) and 1.13 (95% CI 0.42–3.02) in the 65–74-year-old and the >75-year-old subgroups, respectively. The phase III KEYNOTE-048 trial (8) tested pembrolizumab alone or in combination with chemotherapy versus the standard of care as the first-line treatment of R/M HNSCC patients. The authors showed a consistent benefit in patients older than 65 years old in terms of OS both for patients receiving pembrolizumab alone [HR 0.82 (95% CI 0.61–1.10)] and in patients receiving pembrolizumab + chemotherapy [HR 0.55 (95% CI 0.40–0.75)]. In this trial, the median age was 61 years old, with 180 out of a total of 882 patients (20.4%) enrolled with an age more than 65 years old. The phase III JAVELIN-100 trial (18) investigated in LA HNSCC the combination of chemoradiotherapy (CRT) plus avelumab versus CRT alone. The median age was 60 years old, and of the total of 697 patients enrolled, 28.9% were older than 65 years old, of which 102 received avelumab combined with chemoradiotherapy. Data on PFS in elderly patients showed an HR of 1.16 (0.73–1.85), consistent with the HR PFS of the overall population. Regarding durvalumab alone, the OS rates were 73.2% (patients aged 65–74 years old) and 73.3% (patients older than 75 years old) in the EAGLE trial (22) and 90% (patients aged 65–74 years old) and 100% (patients older than 75 years old) in the KESTREL study (21). Data on the combination of durvalumab and tremelimumab for patients older than 65 years old were also reported by Ferris et al. (22) resulting in 82.5% (patients aged 65–74 years old) and 80% (patients older than 75 years old) of OS rates.

### Toxicity

Toxicities are reported in **Table 2**. Of the seven studies included in the systematic review, only the CheckMate 141 trial reported data on toxicities in the older than 65-year-old population (19). Grade 3 to 5 toxicities were reported in 9 out of 68 patients (13%) versus 19 out of 40 patients (47.5%) in the nivolumab and investigator’s choice groups, respectively. In the nivolumab arm, the most common adverse event was skin toxicities (20.6%) (19).

**Table 2:** Adverse events for the 65+ population in the included studies.

Author *Study name*	Intervention (*n*)	G1–5 TRAEs, *n*/*N* (%)	G3–5 TRAEs, *n*/*N* (%)	Serious TRAEs, *n*/*N* (%)	Treatment-related deaths, *n*/*N* (%)
**Saba** *CheckMate 141*	Nivolumab	39/68 (57.4%)^a^	9/68 (13.2%)	NA	2/236 (<1%)
	Investigator’s choice^b^	33/40 (82.5%)	19/40 (47.5%)	NA	1/111 (<1%)

NA, not available; TRAEs, treatment-related adverse events; G, grade; n, number of events; N, number of patients.

a The most common select TRAEs in patients 65+ in the nivolumab arm were skin-related (14 patients: 20.6%).

b IC: treatment of investigator’s choice: docetaxel, methotrexate, or cetuximab.

### Risk of bias and GRADE assessments

Risk of bias and GRADE assessments of the trials included in the meta-analysis were conducted to evaluate the quality of the included studies. Based on the domains of the risk-of-bias tools for non-randomized and randomized trials (ROBINS-1 and ROB-2, respectively), six randomized studies (8, 11, 18, 19, 21, 22) and one non-randomized study (20) were overall considered to be at low risk of bias, although there were some concerns regarding whether age subgroup analysis was pre-planned as pre-protocol in three studies (19, 20, 22). The overall assessment is presented in **Supplementary Table 1** and **Supplementary Figure 1**. The GRADE Working Group grades of evidence are described in **Supplementary Table 2**.

## Discussion

To the best of our knowledge, this is the first systematic review and meta-analysis of prospective trials exploring immunotherapy in elderly head and neck cancer patients. In this regard, no prospective trials focusing on the elderly population are currently available, and high-level evidence is still awaited in this clinical scenario. Commonly, with the exception of the pediatric population, drugs do not undergo registration specifically for particular age groups, allowing elderly patients to receive all standard treatments as long as there are no specific contraindications.

According to our results, elderly and young patients showed a similar benefit from immunotherapy in terms of PFS and OS as their younger counterparts.

In line with the literature, the TOPNIVO study, presented at the ESMO Conference 2020, investigated the safety of nivolumab in elderly (≥70 years) HNSCC patients affected by R/M HNSCC (23). The authors analyzed the subgroup of older (73) and younger patients (270) showing a comparable OS between the two groups (7.9 months versus 7.5 months, for older and younger patients, respectively) (23). Similarly, Saba et al. confirmed better OS and tumor response with nivolumab regardless of age (19). Moreover, when compared with the investigator’s choice (IC), the nivolumab group from the CheckMate 141 trial reported a lower rate of treatment-related adverse events also in the group of elderly patients (19). Referring to the OS subgroup analysis, the authors reported that a different HR for patients aged 65–75 years was 0.57 versus 0.80 of the overall population confirming the beneficial effect of pembrolizumab also in the elderly population (11). According to the phase III EAGLE study, no statistically significant differences in OS were observed for durvalumab with or without tremelimumab versus standard of care (22). Furthermore, Segal et al. in the phase I/II expansion cohort trial confirmed that durvalumab was manageable and safe in HNSCC (20). Likewise, in first-line R/M HNSCC, KEYNOTE-048 did not show a major impact of age on the HR for OS (7, 8). The subgroup analysis of the KEYNOTE-048 trial reported that age did not have a major impact on the HR for OS. When evaluating the data according to CPS ≥1 and CPS ≥20, the HR for death of pembrolizumab versus cetuximab–chemotherapy was also comparable according to age subgroups. Of note, only 36% of the study population was ≥65 years old. Similarly, in the KESTREL study, durvalumab with or without tremelimumab was not superior to the EXTREME regimen in terms of OS in patients who highly expressed PD-L1 (21). Regarding the OS subgroup analysis by age, patients aged <65 and ≥65 years reported similar event rates in durvalumab and EXTREME arms both in the EAGLE and KESTREL trials. Moving to the locally advanced setting, the role of immunotherapy in HNSCC patients is still under investigation. Two phase III trials have investigated the combination of immune checkpoint inhibitors with chemoradiotherapy: the JAVELIN-100 (18) and KEYNOTE-412. The JAVELIN-100 trial randomized 697 patients to CRT plus avelumab for 1 year of CRT or CRT alone. The trial did not show a significant improvement in PFS (HR 1.21, 95% CI 0.93–1.57), which was the primary endpoint. Of note, the subgroup analysis by age reported comparable PFS in younger and older patients. The KEYNOTE-412 trial randomized 804 patients to either CRT plus pembrolizumab or CRT. This trial failed to show a significant increase in event-free survival (EFS) (HR 0.83, 95% CI 0.68–1.03). Also in this trial, the EFS was similar in patients >65 years and younger. The abovementioned trial has not been included as the full report is not yet published. In line with our findings, there is still a significant lack of data regarding TRAEs in older patients receiving immunotherapy. Indeed, only Saba et al. (19) reported a low rate of G3–4 TRAEs in patients older than 65 years receiving nivolumab. Any grade and G3–4 toxicities were similar between patients younger compared with those older than 65 years old: 63.7% versus 57.4% and 16.1% versus 13.2%, respectively. Regarding immune-related adverse events (irAEs) in patients aged 65 years or older, evidence is based mostly on retrospective studies. Neban et al. collected data from 928 elderly patients treated with PD-1 inhibitors: 383 (41%) of them developed an irAE including 113 (12.2%) grade 3–4. Notably, 47 out of 113 (57.6%) were hospitalized for their irAEs (24). Indeed, especially frail patients may be more prone to a complicated course in case of irAE development. A Dutch study reported that frail patients (G8 score < 14) had a higher hospitalization rate, compared with fit patients (G8 score > 14) (25).

As revealed by the pooled data from our systematic review and meta-analysis, elderly patients with R/M HNSCC showed as much benefit to immunotherapy in terms of OS as younger patients. Due to the lack of subgroup analysis of PFS, ORR, and safety by age in the landmark trials, we hypothesize that R/M HNSCC may respond similarly to immunotherapy as younger patients with a manageable toxicity profile. Anti-PD-1 and anti-PD-L1 checkpoint inhibitors have been shown to have better efficacy and lower rate of TRAEs compared with chemotherapy in older patients with bladder cancer, lung cancer, and lymphomas (26). Their benefit has been documented across tumor types. In their meta-analysis, Elias et al. included nine clinical trials on various ICIs in NSCLC, melanoma, RCC, and HNSCC and found that patients >65 years and <65 years had better survival probabilities, with HR of 0.68 and 0.64, respectively (27). Similarly, Nishijima et al. examined nine clinical trials evaluating PD-1 inhibitors or cytotoxic T-lymphocyte-associated protein 4 (CTLA-4) inhibitors in patients with melanoma, NSCLC, and RCC, demonstrating better OS regardless of age, adopting a cutoff age of 65–70 years. The combined HR values for OS in older patients and younger patients who received ICI were 0.73 (*p* < 0.0001) and 0.75 (*p* < 0.0001), respectively, compared with controls (28). Taking into consideration the aforementioned results coming from other disease settings, immunotherapy commonly represents one of the optimal treatment options in clinical practice for elderly R/M HNSCC patients as well.

In 2021, Saleh and colleagues reported a multicenter retrospective analysis on 226 patients older than 70 years old who received immune checkpoint inhibitors (ICIs). It is commonly hypothesized that older and younger adult patients diagnosed with R/M SCCHN treated with ICIs report comparable survival outcomes and toxicity rates (28–30).

Despite the lack of higher-quality evidence-based recommendations, it seems that efficacy can be similar to that observed in younger patients, even though clinical decision-making should take into account the geriatric assessment of elderly patients.

Patients aged ≥70 years with R/M SCCHN receiving ICIs showed similar oncological outcomes in terms of ORR, OS, and PFS when compared with younger patients, with similar toxicity rates (29). However, prospective real-world registers on the role of immunotherapy in the elderly HNSCC setting are awaited.

Our meta-analysis has several limitations, including the restricted number of studies meeting the inclusion criteria and the limited sample of patients in each individual study. Moreover, among the included studies, information regarding the PD-L1, HPV, and ECOG performance status was not available for the elderly subgroup of patients leading to missing conclusions on these specific clinical findings. Indeed, elderly patients are underrepresented in clinical trials, and reporting on elderly subgroups is scarce. In our literature search, 33 studies were excluded because no age-specific outcome data were available (**Figure 1**).

On the other hand, the strength of the current systematic review and meta-analysis is a robust methodology based on a wide search of literature by independent investigators and precise inclusion and exclusion criteria. The novelty of the present paper is the focus on the elderly subgroup of cancer patients receiving immunotherapy which is currently a relevant issue for clinical practice and future research.

In this regard, the practical implication of this systematic review and meta-analysis is the call for action: greater enrollment of older patients in large randomized studies, trials including exclusively elderly patients, meticulous reporting on efficacy and toxicity data, and geriatric assessment outcomes as inclusion criteria or stratification factors. Of note, some large ongoing trials on immunotherapy such as TOPNIVO and KEYNOTE-412 have planned a subgroup analysis by age, and their results are awaited.

## Conclusions

Immunotherapy seems to be effective and well-tolerated in the elderly population, but more data are needed. Because of their favorable toxicity profile, these drugs may be the only anticancer treatment options for many elderly patients, which represent the majority of our cases. Therefore, a call for action is warranted.

## Data availability statement

The raw data supporting the conclusions of this article will be made available by the authors, without undue reservation.

## Author contributions

VS: Writing – review & editing, Writing – original draft, Visualization, Validation, Supervision, Resources, Project administration, Methodology, Investigation, Funding acquisition, Formal Analysis, Data curation, Conceptualization. SC: Writing – review & editing, Writing – original draft, Methodology. MSc: Methodology, Writing – review & editing, Writing – original draft. MSa: Methodology, Writing – review & editing, Writing – original draft. HB: Conceptualization, Writing – review & editing, Writing – original draft. PB: Conceptualization, Writing – review & editing, Writing – original draft. FC: Data curation, Writing – review & editing, Writing – original draft. LL: Data curation, Writing – review & editing, Writing – original draft. MO: Data curation, Writing – review & editing, Writing – original draft. MU: Conceptualization, Writing – review & editing, Writing – original draft. PS: Visualization, Validation, Supervision, Software, Resources, Project administration, Methodology, Investigation, Funding acquisition, Formal Analysis, Data curation, Conceptualization, Writing – review & editing, Writing – original draft.
