# Supplementary material for: Immunotherapy in elderly head and neck cancer patients: a systematic review and meta-analysis
Source: Front Oncol. 2024 May 10;14:1395838. doi: 10.3389/fonc.2024.1395838 (PMC11127588; doi:10.3389/fonc.2024.1395838)
Supplement: Supplementary file 1 [file Table_1.docx]

| **Supplemental Table 1** Risk of Bias assessment (randomized included studies) | | | | | | | | | | | |
| --- | --- | --- | --- | --- | --- | --- | --- | --- | --- | --- | --- |
| **Intention-to-treat** | **Study ID** | **Experimental** | **Comparator** | **Outcome** | **Weight** | **D1** | **D2** | **D3** | **D4** | **D5** | **Overall** |
|  | Burtness, 2019 | Pembrolizumab | Cetuximab-CT | OS and PFS | 1 |  |  |  |  |  |  |
|  | Ferris, 2018 | Nivolumab | IC Chemotherapy | OS and PFS | 1 |  |  |  |  |  |  |
|  | Cohen, 2019 | Pembrolizumab | IC chemotherapy | OS | 1 |  |  |  |  |  |  |
|  | Lee, 2021 | Avelumab-CRT | Placebo-CRT | PFS | 1 |  |  |  |  |  |  |
|  | Ferris, 2020 | Durva, Durva-Treme | Investigator's choice CT | OS | 1 |  |  |  |  |  |  |
|  | Psyrri, 2023 | Durva, Durva-Treme | Cetuximab-CT | OS and PFS | 1 |  |  |  |  |  |  |

| **Legend** | | | |  |  |
| --- | --- | --- | --- | --- | --- |
| \|  \| \| --- \| | Low risk | D1 | Randomisation process |  |  |
| \|  \| \| --- \| | Some concerns | D2 | Deviations from the intended interventions |  |  |
| \|  \| \| --- \| | High risk | D3 | Missing outcome data |  |  |
|  |  | D4 | Measurement of the outcome |  |  |
|  |  | D5 | Selection of the reported result |  |  |
