# Supplementary material for: Immunotherapy in elderly head and neck cancer patients: a systematic review and meta-analysis
Source: Front Oncol. 2024 May 10;14:1395838. doi: 10.3389/fonc.2024.1395838 (PMC11127588; doi:10.3389/fonc.2024.1395838)
Supplement: Supplementary file 2 [file Table_2.docx]

**Supplemental Table 2.** GRADE approach to assess certainty.

|  | **N° of 65+ y patients, (N° of studies)** | **Quality of the evidence (GRADE)** |
| --- | --- | --- |
| **Overall survival (65+ y patients)** | 456 (3) | ⊕⊕⊕⊕  **High** (randomized trials, low risk of bias and low rate of heterogeneity) |
| **Progression-free survival (65+ y patients)** | 315 (2) | ⊕⊕⊕  **Moderate** (randomized trials, low risk of bias and moderate rate of heterogeneity) |
