# Supplementary material for: Immunotherapy in elderly head and neck cancer patients: a systematic review and meta-analysis
Source: Front Oncol. 2024 May 10;14:1395838. doi: 10.3389/fonc.2024.1395838 (PMC11127588; doi:10.3389/fonc.2024.1395838)
Supplement: Supplementary file 3 [file DataSheet_1.pdf]

Methods

Search Strategy

A comprehensive literature search of PubMed/MEDLINE, COCHRANE and EMBASE databases was conducted. The literature search was performed in December 2022 (2000 to December 2022) using the keywords "recurrent; metastatic; locally; advanced; head and neck cancer; oral cavity; pharynx; oropharynx; hypopharynx; larynx; immunotherapy; immune checkpoint inhibitor". The review followed the Preferred Reporting Items for Systematic Reviews and Meta-Analyses (PRISMA) Statement [16].

Data selection

Titles and abstracts were screened by two independent researchers (VS and MS) after removing duplicates. Original articles, reviews and meta-analyses read in full text were checked for relevant references to be included.

<u>Inclusion Criteria (all must be met):</u>

- Original prospective studies (or relevant updates thereof) that investigate the role of immune checkpoint inhibitors in elderly patients ($\geq$ 65 years) with previously untreated LA-HNSCC of the oral cavity, oropharynx, hypopharynx, or larynx OR with R/M-HNSCC of the oral cavity, oropharynx, hypopharynx, or larynx as first- or second-line palliative treatment.
- Efficacy outcomes OR side effects OR quality of life aspects measured as primary OR secondary outcomes and reported either as part of the main study analysis OR a subgroup analysis.
- English language
- Full-text or abstract available
- Human subjects

<u>Exclusion Criteria:</u>

- Studies enrolling >25% of patients with nasopharyngeal carcinoma or cancer of unknown origin
- Editorials, commentaries, letters and conference abstracts not presenting original data except for relevant study updates
- Retrospective analyses

Across the articles identified in the initial systematic review, if studies were phase II/III randomized controlled trials (RCTs) and met the inclusion criteria they were included in the meta-analysis. Moreover, studies were eligible for meta-analysis if they provided a hazard ratio (HR) and a corresponding measure of statistical uncertainty (e.g. 95% confidence intervals (CI), standard errors, variance, or exact p-values).

Data extraction

Data extraction was completed by two (FC and MS) independent reviewers to ensure consistency and accuracy. FC and MS entered the data extracted from each eligible study into an internally piloted spreadsheet. Disagreements were managed and solved by iteration, discussion and consensus among the other co-authors. Data was collected on the patient and treatment characteristics (e.g. cancer site, setting of disease, type of immunotherapy drug, combination treatment) and the study design. Outcomes and findings were collected as categorical variables (i.e. event proportions) and/or continuous variables (i.e. mean scores). Data from the intent-to-treat and per-protocol analyses was analyzed where available. Subgroup analyses were performed if applicable.

Outcomes

The co-primary endpoints of the present analysis were the OS and the PFS of elderly patients with LA and RM HNSCC of the oral cavity, oropharynx, hypo-pharynx, or larynx. We defined as elderly population as the subgroup of patients that aged 65 years or older. The secondary endpoints of the

meta-analysis were safety of the single-agent immunotherapy and health-related quality of life (HRQoL) of elderly subgroup.

Statistical Analysis

Meta-analysis was conducted from at least two independent papers for a given endpoint. Study-specific estimates of association have been pooled into a summary estimate using random effects meta-analysis models, and results have been visualized using forest plots. The heterogeneity across studies will be quantified using the I2 statistics, and if this exceeds 50%, meta-regression, subgroup analysis, and leave-one-out sensitivity analysis will be carried out to explore sources of heterogeneity. Finally, the presence of publication bias will be searched for graphically using funnel plots, and by means of Egger's and Begg's tests. In case significant publication bias is detected, the trim-and-fill method will be applied to input missing estimates and calculate an adjusted summary estimate.

Assessment of risk of bias and certainty of included studies

We adopted the Cochrane tools to assess the risk of bias for randomized and non randomized controlled trials. Bias is assessed as a judgment (high, low, or unclear) for individual elements concerning selection, performance, attrition, reporting, and other [17]. Two independent reviewers (MOB and LL) assessed the risk of bias of each trial and a third author (VS) was consulted in case of disagreements. We used GRADE (Grading of Recommendations, Assessment, Development and Evaluations) strategy [18] to assess certainty in the body of evidence for an outcome.
