# Supplementary material for: Immunotherapy in elderly head and neck cancer patients: a systematic review and meta-analysis
Source: Front Oncol. 2024 May 10;14:1395838. doi: 10.3389/fonc.2024.1395838 (PMC11127588; doi:10.3389/fonc.2024.1395838)
Supplement: Supplementary file 4 [file Image_1.pdf]

The Risk Of Bias In Non-randomized Studies – of Interventions (ROBINS-I) assessment tool

(version for cohort-type studies)

Version 19 September 2016

This work is licensed under a Creative Commons Attribution-NonCommercial-NoDerivatives 4.0 International License.

ROBINS-I tool (Stage I): At protocol stage

Specify the review question

Participants	62
Experimental intervention	Durvalumab
Comparator	NA
Outcomes	Safety; Activity

List the confounding domains relevant to all or most studies

List co-interventions that could be different between intervention groups and that could impact on outcomes

ROBINS-I tool (Stage II): For each study

Specify a target randomized trial specific to the study

Design	Individually randomized / Cluster randomized / Matched (e.g. cross-over)
Participants	NA
Experimental intervention	NA
Comparator	NA

Is your aim for this study…?

X to assess the effect of *assignment to* intervention

☐ to assess the effect of *starting and adhering to* intervention

Specify the outcome

Specify which outcome is being assessed for risk of bias (typically from among those earmarked for the Summary of Findings table). Specify whether this is a proposed benefit or harm of intervention.

Activity

Specify the numerical result being assessed

In case of multiple alternative analyses being presented, specify the numeric result (e.g. RR = 1.52 (95% CI 0.83 to 2.77) and/or a reference (e.g. to a table, figure or paragraph) that uniquely defines the result being assessed.

RR 6.5% (95% CI 1.8-1.57)

Risk of bias assessment

Responses underlined in green are potential markers for low risk of bias, and responses in red are potential markers for a risk of bias. Where questions relate only to sign posts to other questions, no formatting is used.

Signalling questions	Description	Response options
**Bias due to confounding**		
1.1 Is there potential for confounding of the effect of intervention in this study?<br>**If N/PN to 1.1:** the study can be considered to be at low risk of bias due to confounding and no further signalling questions need be considered		Y / PY / PN / N
**If Y/PY to 1.1**: determine whether there is a need to assess time-varying confounding:		
1.2. Was the analysis based on splitting participants' follow up time according to intervention received?<br>**If N/PN**, answer questions relating to baseline confounding (1.4 to 1.6)<br>**If Y/PY**, go to question 1.3.		NA / Y / PY / PN / N / NI
1.3. Were intervention discontinuations or switches likely to be related to factors that are prognostic for the outcome?<br>**If N/PN**, answer questions relating to baseline confounding (1.4 to 1.6)<br>**If Y/PY**, answer questions relating to both baseline and time-varying confounding (1.7 and 1.8)		NA / Y / PY / PN / N / NI

**Questions relating to baseline confounding only**		
1.4. Did the authors use an appropriate analysis method that controlled for all the important confounding domains?		NA / Y / PY / PN / N / NI
1.5. **If Y/PY to 1.4**: Were confounding domains that were controlled for measured validly and reliably by the variables available in this study?		NA / Y / PY / PN / N / NI
1.6. Did the authors control for any post-intervention variables that could have been affected by the intervention?		NA / Y / PY / PN / N / NI
**Questions relating to baseline and time-varying confounding**		
1.7. Did the authors use an appropriate analysis method that controlled for all the important confounding domains and for time-varying confounding?		NA / Y / PY / PN / N / NI
1.8. **If Y/PY to 1.7**: Were confounding domains that were controlled for measured validly and reliably by the variables available in this study?		NA / Y / PY / PN / N / NI
**Risk of bias judgement**		Low / Moderate / Serious / Critical / NI
Optional: What is the predicted direction of bias due to confounding?		Favours experimental / Favours comparator / Unpredictable

Bias in selection of participants into the study		
2.1. Was selection of participants into the study (or into the analysis) based on participant characteristics observed after the start of intervention?<br>**If N/PN to 2.1:** go to 2.4		Y / PY / PN / N / NI
2.2. **If Y/PY to 2.1**: Were the post-intervention variables that influenced selection likely to be associated with intervention?		NA / Y / PY / PN / N / NI
2.3 **If Y/PY to 2.2**: Were the post-intervention variables that influenced selection likely to be influenced by the outcome or a cause of the outcome?		NA / Y / PY / PN / N / NI
2.4. Do start of follow-up and start of intervention coincide for most participants?		Y / PY / PN / N / NI
2.5. **If Y/PY to 2.2 and 2.3, or N/PN to 2.4**: Were adjustment techniques used that are likely to correct for the presence of selection biases?		NA / Y / PY / PN / N / NI
**Risk of bias judgement**		Low / Moderate / Serious / Critical / NI
Optional: What is the predicted direction of bias due to selection of participants into the study?		Favours experimental / Favours comparator / Towards null /Away from null / Unpredictable

Bias in classification of interventions		
3.1 Were intervention groups clearly defined?		Y / PY / PN / N / NI
3.2 Was the information used to define intervention groups recorded at the start of the intervention?		Y / PY / PN / N / NI
3.3 Could classification of intervention status have been affected by knowledge of the outcome or risk of the outcome?		Y / PY / PN / N / NI
**Risk of bias judgement**		Low / Moderate / Serious / Critical / NI
Optional: What is the predicted direction of bias due to classification of interventions?		Favours experimental / Favours comparator / Towards null /Away from null / Unpredictable

Bias due to deviations from intended interventions		
**If your aim for this study is to assess the effect of assignment to intervention, answer questions 4.1 and 4.2**		
4.1. Were there deviations from the intended intervention beyond what would be expected in usual practice?		Y / PY / PN / N / NI
4.2. **If Y/PY to 4.1**: Were these deviations from intended intervention unbalanced between groups *and* likely to have affected the outcome?		NA / Y / PY / PN / N / NI
**If your aim for this study is to assess the effect of starting and adhering to intervention, answer questions 4.3 to 4.6**		
4.3. Were important co-interventions balanced across intervention groups?		Y / PY / PN / N / NI
4.4. Was the intervention implemented successfully for most participants?		Y / PY / PN / N / NI
4.5. Did study participants adhere to the assigned intervention regimen?		Y / PY / PN / N / NI
4.6. **If N/PN to 4.3, 4.4 or 4.5**: Was an appropriate analysis used to estimate the effect of starting and adhering to the intervention?		NA / Y / PY / PN / N / NI
**Risk of bias judgement**		Low / Moderate / Serious / Critical / NI
Optional: What is the predicted direction of bias due to deviations from the intended interventions?		Favours experimental / Favours comparator / Towards null /Away from null / Unpredictable

Bias due to missing data		
5.1 Were outcome data available for all, or nearly all, participants?		Y / PY / PN / N / NI
5.2 Were participants excluded due to missing data on intervention status?		Y / PY / PN / N / NI
5.3 Were participants excluded due to missing data on other variables needed for the analysis?		Y / PY / PN / N / NI
5.4 **If PN/N to 5.1, or Y/PY to 5.2 or 5.3**: Are the proportion of participants and reasons for missing data similar across interventions?		NA / Y / PY / PN / N / NI
5.5 **If PN/N to 5.1, or Y/PY to 5.2 or 5.3**: Is there evidence that results were robust to the presence of missing data?		NA / Y / PY / PN / N / NI
**Risk of bias judgement**		Low / Moderate / Serious / Critical / NI
Optional: What is the predicted direction of bias due to missing data?		Favours experimental / Favours comparator / Towards null /Away from null / Unpredictable

Bias in measurement of outcomes		
6.1 Could the outcome measure have been influenced by knowledge of the intervention received?		Y / PY / PN / N / NI
6.2 Were outcome assessors aware of the intervention received by study participants?		Y / PY / PN / N / NI
6.3 Were the methods of outcome assessment comparable across intervention groups?		Y / PY / PN / N / NI
6.4 Were any systematic errors in measurement of the outcome related to intervention received?		Y / PY / PN / N / NI
**Risk of bias judgement**		Low / Moderate / Serious / Critical / NI
Optional: What is the predicted direction of bias due to measurement of outcomes?		Favours experimental / Favours comparator / Towards null /Away from null / Unpredictable

Bias in selection of the reported result		
Is the reported effect estimate likely to be selected, on the basis of the results, from...<br>7.1. ... multiple outcome *measurements* within the outcome domain?		Y / PY / PN / N / NI
7.2 ... multiple *analyses* of the intervention-outcome relationship?		Y / PY / PN / N / NI
7.3 ... different *subgroups*?		Y / PY / PN / N / NI
**Risk of bias judgement**		Low / Moderate / Serious / Critical / NI
Optional: What is the predicted direction of bias due to selection of the reported result?		Favours experimental / Favours comparator / Towards null /Away from null / Unpredictable

Overall bias		
**Risk of bias judgement**		Low / Moderate / Serious / Critical / NI
Optional: What is the overall predicted direction of bias for this outcome?		Favours experimental / Favours comparator / Towards null /Away from null / Unpredictable

This work is licensed under a Creative Commons Attribution-NonCommercial-NoDerivatives 4.0 International License.
